# 95349 T1-T4 in 3 (Minutes)

**DOI:** 10.1017/cts.2021.514

**Published:** 2021-03-30

**Authors:** Sharon Croisant, Alisha Goldberg, John Prochaska, Chantele Singleton, Krista Bohn, Lance Hallberg

**Affiliations:** University of Texas Medical Branch

## Abstract

ABSTRACT IMPACT: The purpose of the T1-T4 in 3 Minutes program is to improve trainees’ capacity for communication of complex to a non-scientific audience as well as to ensure that our community stakeholders have access to, and understanding of, ongoing clinical and translation research OBJECTIVES/GOALS: The T1-T4 in 3 Program: ο Increases knowledge of research across institution; ο Increases capacity of trainees to convey complex science to lay audiences, funders, colleagues, and the media; ο Increases health and scientific literacy; ο Bridges gaps between trainees and potential entrepreneurial mentors METHODS/STUDY POPULATION: T1-T4 in 3 (Minutes) is an adaptation of the University of Queensland’s Three Minute Thesis competition in which PhD students present their thesis in 3 minutes or less to a lay audience. The competition enables them to cogently communicate their ideas and research findings to a non-specialist audience. Our adapted version, T1-T4 in 3, requires a presentation in three minutes or less to a lay audience, but rather than a thesis, the topics are on trainees’ research, and in this particular case, an idea for a commercial venture. The competition provides awards for the first- and second-best projects as determined by a panel of judges, and a ‘people’s choice’ award determined by a lay audience. RESULTS/ANTICIPATED RESULTS: This exercise is anticipated to improve trainees’ capacity for communications as well as ensure that community stakeholders and research and business community partners have access to, and understanding of, ongoing clinical and translation research with potential commercial applications. Further, the increased ability of our faculty and trainees to effectively communicate complex science to the public and other audiences’‘ including potential funders’‘ supports additional stakeholder dissemination mechanisms by increasing their confidence in their abilities to converse with non-specialists about their research, thus increasing the likelihood of participation in other community-based activities. DISCUSSION/SIGNIFICANCE OF FINDINGS: To increase ITS commercialization efforts, we envision involving numerous external partners to educate, fund, and support new ventures. T1-T4 in 3 judges will include commercialization scholars from regional and national institutions as well as pharmaceutical entities and regional angel investors.

